# High ovarian hormones present during fear extinction reduce fear relapse through a nigrostriatal dopamine pathway

**DOI:** 10.1186/s13293-025-00722-7

**Published:** 2025-06-01

**Authors:** Alyssa A. Hohorst, Margaret K. Tanner, Rebecca Han, Kamryn M. Korth, Jessica D. Westerman, Carolina Sanchez Mendoza, Miles Q. Dryden, Lareina A. Alvarez, Remla A. Abdul, Esteban C. Loetz, Erik B. Oleson, Benjamin N. Greenwood

**Affiliations:** 1 Department of Psychology, University of Colorado Denver, Denver, CO USA; 2 Department of Integrative Biology, University of Colorado Denver, Denver, CO USA; 3 Biological Sciences Division, University of California San Diego, San Diego, CA USA; 4 School of Medicine, Vanderbilt University, Nashville, TN USA

**Keywords:** Estrous cycle, Estrogen, Dopamine, Fear extinction, Fear renewal, Spontaneous recovery, Substantia nigra, Dorsolateral striatum

## Abstract

**Background:**

Elevated ovarian hormones during fear extinction can enhance fear extinction memory retention and reduce fear renewal, but the mechanisms remain unknown. High levels of ovarian hormones are associated with heightened dopamine (DA) transmission, a key player in fear extinction. In males, stimulation of substantia nigra (SN) DA neurons during fear extinction reduces renewal; an effect mimicked by DA D1 receptor agonist administration into the dorsolateral striatum (DLS), a primary target of the SN. The current studies tested the role of the SN-DLS pathway in estrous cycle-modulation of fear extinction and relapse.

**Methods:**

Male and female Long-Evans rats were used to investigate the effects of sex and ovarian hormone levels during fear extinction on later fear relapse and underlying mechanisms. Fear extinction-induced cFos in SN DA neurons was quantified with double-label immunohistochemistry. An intersectional chemogenetic approach was used to determine whether SN-DLS pathway activity during fear extinction is necessary and sufficient for observed effects of ovarian hormones on fear relapse. Finally, fast scan cyclic voltammetry revealed the effects of sex and ovarian hormones on electrically-evoked DA release in the DLS and verified the effectiveness of chemogenetic approaches.

**Results:**

Female rats exposed to fear extinction during proestrus or estrus (Pro/Est; high hormones) had less relapse (renewal and spontaneous recovery) compared to males or females exposed to fear extinction during metestrus or diestrus (Met/Di; low hormones). Fear extinction-induced cFos within SN DA neurons and electrically-evoked DA release in the DLS was highest in female rats during Pro/Est. The behavioral and neurochemical effects of Pro/Est were mimicked by estradiol administration to ovariectomized female rats. Inhibition of the SN-DLS pathway suppressed electrically-evoked DA release in the DLS and restored fear renewal in females exposed to simultaneous fear extinction and SN-DLS inhibition during Pro/Est. Conversely, stimulation of the SN-DLS pathway during extinction reduced fear renewal in males.

**Conclusions:**

Results indicate that ovarian hormones present during fear extinction reduce later fear relapse through a SN-DLS dopamine pathway. Data suggest the SN-DLS DA pathway is a novel target for the reduction of fear relapse in both sexes.

**Supplementary Information:**

The online version contains supplementary material available at 10.1186/s13293-025-00722-7.

## Plain Language summary

Women are at higher risk than men for common psychiatric disorders such as post-traumatic stress disorder. Exposure therapy is used to treat these disorders whereby subjects learn that cues previously paired with trauma no longer predict danger. However, the efficacy of exposure therapy is limited by the return of fear (relapse), even after successful fear extinction. Research is needed to better understand sex differences in the processes governing fear extinction learning and relapse. Levels of ovarian hormones present during fear extinction can influence the strength of fear extinction memory and relapse, but the mechanisms underlying the effect of ovarian hormones remain unknown. Due to an emerging role for dopamine (DA) and the substantia nigra-to-dorsolateral striatum (SN-DLS) DA pathway in relapse-resistant fear extinction, we investigated the involvement of the SN-DLS DA pathway in ovarian hormone-modulation of fear extinction and relapse. We observe that females with elevated ovarian hormones during fear extinction have less fear relapse compared to males and females with low ovarian hormones, an effect attributable to estradiol. Elevated ovarian hormones potentiated DA neural activity during fear extinction in the SN and DA release in the DLS, while inhibition of the SN-DLS pathway during fear extinction restores fear relapse in females exposed to fear extinction in the presence of high ovarian hormones. Additionally, stimulation of the SN-DLS pathway during fear extinction reduces relapse in males. The results of this study implicate the SN-DLS pathway as a novel target for the reduction of relapse in both sexes.

**Supplementary Information:**

The online version contains supplementary material available at 10.1186/s13293-025-00722-7.

## Highlights

High levels of ovarian hormones during fear extinction is associated with low levels of fear relapse in adult, female rats.

Electrically-evoked dopamine release in the dorsolateral striatum is potentiated by estradiol and is highest in phases of the estrous cycle characterized by high levels of ovarian hormones.

Activity of substantia nigra dopamine neurons during fear extinction is greatest in females exposed to fear extinction during estrous cycle phases associated with high levels of ovarian hormones.

Inhibition of the substantia nigra-to-dorsolateral striatum pathway during fear extinction restores fear relapse in females exposed to extinction during conditions of high ovarian hormones.

Stimulation of the substantia nigra-to-dorsolateral striatum pathway during fear extinction reduces fear relapse in males.

**Supplementary Information:**

The online version contains supplementary material available at 10.1186/s13293-025-00722-7.

## Introduction

Fear extinction memories remain susceptible to fear relapse phenomena such as renewal (return of fear in contexts different from where extinction took place) and spontaneous recovery (return of fear after the passage of time; [1, 2]). Although means to enhance fear extinction through manipulation of known extinction circuits have been identified, prior work has had limited success in reducing fear relapse in a clinical setting. Novel strategies to reduce fear relapse after fear extinction are needed.

High levels of ovarian hormones present at the time of fear extinction can enhance fear extinction retention [3, 4, 5, 6, 7]; thus, ovarian hormones could also potentially impact fear relapse. However, data on the effects of sex on fear relapse are mixed. One study reported a lack of fear renewal in cycling females [8] and another found no sex differences in renewal [9]. Neither prior study assessed levels of ovarian hormones present at the time of extinction. Bouchet et al. (2017) reported that female rats exposed to extinction during reproductive cycle phases during which estrogen is elevated (proestrus and estrus; Pro/Est) have less fear renewal compared to low estrogen phases (metestrus and diestrus, Met/Di; [10]). While these data suggest that high levels of ovarian hormones during fear extinction can reduce fear renewal, it remains unknown how renewal in Pro/Est and Met/Di females compare to males, whether estrous phase during extinction impacts other forms of relapse, and which neural mechanisms are involved.

Dopamine (DA) is emerging as a key player in fear extinction [11, 12, 13]. Importantly, augmenting fear extinction with DA agonists can render extinction memories resistant to fear relapse. For example, the DA agonist L-DOPA administered after fear extinction reduces the renewal and spontaneous recovery of conditioned fear in male mice and humans [14, 15]. Elevated estrogen, either via 17β-estradiol benzoate (E2) administration [16] or during Pro/Est [17, 18], is associated with potentiated stimulus-evoked DA release. It is therefore possible that high levels of ovarian hormones during fear extinction renders extinction memory resistant to fear relapse through a mechanism involving DA.

Unique roles of ascending DA pathways in fear extinction are being uncovered. The mesocorticolimbic pathway, originating in the ventral tegmental area and projecting to forebrain regions classically implicated in fear extinction, supports the acquisition and consolidation of extinction [11, 12, 13, 19], but whether alterations in DA transmission in these regions impacts fear relapse is not well understood. The nigrostriatal pathway, consisting of midbrain DA neurons arising from the substantia nigra (SN) and projecting to the dorsal striatum (DS), is increasingly implicated in behaviors beyond its canonical role in movement [20, 21, 22, 23, 24, 25, 26]. Consistent with a role for nigrostriatal DA in fear relapse, we have found that chemogenetic stimulation of SN DA neurons during fear extinction prevents fear renewal in male rats [27]. These results are particularly interesting given that females are reported to have potentiated stimulus-evoked DA release in the DS when in Est compared to Di [17]. SN DA neurons target both the dorsolateral (DLS) and dorsomedial (DMS) subregions of the DS, which subserve unique functions. The effect of SN DA stimulation during fear extinction on fear renewal in males is mimicked by a D1 receptor agonist injected into the DLS, but not the DMS [28]. These data support the hypothesis that activity of a SN-DLS pathway during fear extinction mediates the effects of ovarian hormones on fear relapse.

The current studies investigated the role of the SN-DLS pathway in estrous cycle-modulation of fear extinction and relapse. We observe that females undergoing fear extinction in Pro/Est have less fear renewal and spontaneous recovery compared to males and females in Met/Di, an effect replicated by E2 administration. High levels of ovarian hormones are associated with potentiated activity of the SN-DLS pathway, inhibition of which during fear extinction restores fear relapse in females exposed to extinction during Pro/Est, and stimulation of which reduces relapse in males. Results provide new insight into how the estrous cycle interacts with the nigrostriatal pathway to render fear extinction memory resistant to fear relapse and implicate the SN-DLS pathway as a novel target for the reduction of relapse in both sexes.

## Materials and methods

### Animals and housing

100 male and 172 female (P56 on arrival) Long-Evans rats (Charles River, Wilmington, MA) were pair-housed in ventilated cages (24 L x 45.4 W x 21 H cm) in a temperature- (22° C) and humidity-controlled vivarium accredited by the Association for Assessment and Accreditation of Laboratory Animal Care located on the University of Colorado Denver Auraria campus. Food (Teklad 2020X rodent diet, Envigo) and water were available *ad libitum*. All experimental protocols were approved by the University of Colorado Denver Animal Care and Use Committee.

### Estrous phase identification

Vaginal lavages were collected for 7 consecutive days prior to the start of behavioral procedures to establish cycle and habituate rats to lavage. Using a sterile, blunt-tipped eye dropper filled with ~ 0.5 mL sterile-filtered 0.2% PBS-Brij solution (Brij 35 Solution 30%; Sigma Aldrich, B4184), the vagina was gently flushed, and the collected fluid was transferred onto a microscope slide for visualization under a 20X objective lens (Olympus BX53), as previously described [29]. Vaginal lavages were also collected ~ 2 h prior to, and immediately after, each behavioral test to ensure correct phase identification during behavioral tests. Males were handled for an equivalent period.

### Surgical procedures

All surgery was performed under Ketamine (75.0 mg/kg, i.p.) and Medetomidine (0.5 mg/kg, i.p.) anesthesia. Injections of Carprofen (5 mg/kg, s.c.) and Penicillin G (22,000 IU/rat s.c.) were administered at induction and every 24 h for 72 h after surgery and rats recovered for at least 2 weeks prior to experimentation. Bilateral ovariectomy (OVX, *n* = 23), was performed as previously described (Supplemental materials; [30]). Stereotaxic viral infusions were administered bilaterally via Hamilton needles connected to infusion pumps (World Precision Instruments, Sarasota, FL) at a rate of 0.1 µL/minute. All rats used in chemogenetic studies received bilateral infusion of AAV2/retro-eSYN-EGFP-T2A-iCre-WPRE (Vector Biolabs, Malvern, PA Cat# VB4855; [31]) into the DLS (+ 0.5 mm anterior, ± 3.9 mm lateral, -5.4 mm ventral from the top of the skull) and either pAAV8-hSyn-DIO-mCherry (mCherry; gift from Bryan Roth; Addgene plasmid # 50459; http://n2t.net/addgene:50459), pAAV8-hSyn-DIO-hM4Di(G_i_)-mCherry (G_i_-DREADD; gift from Bryan Roth; Addgene plasmid # 44362; http://n2t.net/addgene:44362; RRID: Addgene_44362; [32]), or pAAV8-hSyn-DIO-hM3D(G_q_)-mCherry (G_q_-DREADD; gift from Bryan Roth; Addgene plasmid # 44361; http://n2t.net/addgene:44361; RRID: Addgene_44361; [32]) into the SN (− 5.4 mm anterior, ± 3.0 mm lateral, − 8.4 mm ventral from the top of the skull). Viral transfection in the DLS and SN was verified in all rats by inspection of viral expression (Olympus BX53; Center Valley, PA). Immunohistochemistry (IHC) was used to amplify mCherry terminal expression and levels of mCherry were compared between brain regions using densitometry (Supplemental Materials). Rats with missed viral injections or undetectable levels of viral expression were excluded.

### Drugs

Rats received saline (1 mL/kg, i.p.) or JHU37160 dihydrochloride (J60, Hello Bio, Princeton, NJ, Cat # HB6261, 0.1 mg/kg, i.p.) 30 min prior to fear extinction, fast scan cyclic voltammetry (FSCV), and/or locomotion procedures. J60 was dissolved immediately before use in sterile saline. E2 (Sigma Aldrich, St. Louis, MO, Cat# E8875-1G, 4.5 µg/0.1 mL) was dissolved immediately before use in sesame oil (Veh; Sigma Aldrich, Cat# S3547). Starting 5 days following OVX, all OVX rats received 1 subcutaneous injection of E2 every 4 days for 2 weeks before behavioral testing. Four days after the last cyclic E2 injection, OVX rats were randomly assigned to receive either E2 or Veh (0.1 mL/kg, s.c.) 30 min prior to fear extinction or FSCV testing.

### Conditioned fear behavior

Auditory fear conditioning (4 CS-US presentations; 10 s, 80 dB, 2 kHz auditory CS terminating with a 1 s, 0.8 mA foot shock US, 1 min ITI) took place in context A and auditory fear extinction (20 CS; 1 min ITI) occurred 24 h later in context B. Auditory fear conditioning was used to avoid potential sex differences in conditioning, which have been observed with contextual fear conditioning [33, 34]. The day after fear extinction, rats were re-exposed to repeated presentations of the CS (5–20 CS, depending on experiment; 1 min ITI) in the same context as where extinction took place (context B), or a different context (context C). Rats were assigned to context B or context C based on freezing levels during extinction to avoid arbitrary differences in extinction levels between groups. Spontaneous recovery testing took place in context B 1 week later. Contexts A, B, and C differed in shape, odor, lighting, and textures as previously described ([27]; Supplementary Materials). All behavioral tests were recorded with overhead cameras, and videos were scored by both EthoVision XT (Leesburg, VA) and an experimenter blind to treatment conditions.

### Locomotor activity

Since conditioned freezing can be influenced by locomotor activity as well as fear, locomotor activity was assessed during the 3 min period prior to the first CS during each behavioral test using Noldus. Additionally, because females have been reported to express fear with high-velocity, darting behavior [35], high-velocity movement throughout all behavioral tests were measured using Noldus. Similar to prior reports [36], no appreciable (mean < 1% during all tests) high-velocity movement was detected during behavioral tests, so these data were not analyzed. The effects of chemogenetic manipulations on locomotor activity was again assessed 24 h following behavioral testing. Rats were placed into locomotor activity chambers (17″ × 17″ × 12″ L x W x H; Med Associates, Fairfax, VT), which use beam breaks to calculate the average distance traveled over 1 h. J60 was administered 30 min prior to testing on day 1 only.

### Fast scan Cyclic voltammetry

Effects of sex/estrous phase (*n* = 18), E2 administration (*n* = 4), and chemogenetic manipulations (*n* = 33) on DS DA release were evaluated using FSCV in subsets of rats used in behavioral experiments. Under urethane (1.5 g/kg, i.p.) anesthesia, rats were placed in stereotaxic frames and 3–4 holes were drilled into the skull. A glass capillary-encased carbon fiber microelectrode (carbon fiber extending 120–150 μm beyond glass capillary) was lowered approximately 4.5–5.2 mm (from dura) into either the DMS (+ 0.5 mm anterior, ± 1.8 mm lateral) or DLS (+ 0.5 mm anterior, ± 3.9 mm lateral from the top of the skull). A stimulating electrode (Plastics One, Roanoke, VA) was lowered 7.2–7.4 mm (from dura) into the SN (− 5.4 mm anterior, ± 3.0 mm lateral, from the top of the skull) and an Ag/AgCl reference electrode was implanted into the contralateral hemisphere just below the surface of the skull. Voltammetric recordings were conducted by applying a triangular waveform (− 0.4 V to 1.3 V; 400 V/s) which allowed for the detection of DA from cyclic voltammograms taken every 100 ms. To increase electrode sensitivity, the waveform was first applied at 60 Hz for ~ 30 min, but was reduced to 10 Hz before experimentation. Recordings were taken 30 min after Veh or saline administration to establish a baseline response to electrical stimulation. 30 min following E2 or J60 administration, data were collected in three 15 s recordings taken every 5 min. Recordings were collected again 1 h after E2 administration. Biphasic stimulation of the SN (24 rectangular pulses, 60 Hz, 300 µA) was delivered via the stimulating electrode 5 s into each recording. Concentration was calculated from data collected during the recordings using linear regression and principle component regression as previously described [37].

### Tyrosine hydroxylase and cFos double immunohistochemistry

Male (*n* = 8) and cycling female rats (Met/Di *n* = 7; Pro/Est *n* = 11) were exposed to fear conditioning followed 24 h later by fear extinction. Females were grouped according to estrous phase during fear extinction. Rats were trans-cardially perfused with ice-cold saline and 4% paraformaldehyde 90 min after fear extinction. Double-label tyrosine hydroxylase (TH) and cFos IHC and quantification occurred according to previously published protocols [38].

### Statistical analysis

Percent time spent freezing was calculated by averaging data from the blind experimenter and the Noldus scores. Group differences in freezing and movement prior to the first CS in all contexts were analyzed with ANOVA. Group differences in freezing during fear conditioning were analyzed with repeated measures ANOVA. Freezing during fear extinction was collapsed into 10 blocks consisting of 2 CS each, and group differences were analyzed with repeated measures ANOVA. Freezing across all trials during fear renewal and spontaneous recovery were averaged and group differences were compared with ANOVA. Group differences in locomotor activity, DA concentration relative to Veh or saline, and % of TH-positive cells containing cFos were analyzed with ANOVA. The Shapiro-Wilk and Brown-Forsythe tests verified normality and equal variance of the data, respectively, prior to running ANOVAs. Effect sizes are reported as partial eta squared (n^2^p). Grubb’s tests were used to determine statistical outliers. Bonferroni post-hoc analyses were performed when appropriate. Group differences were considered significant when *p* < 0.05.

## Results

### Sex and estrous phase during fear extinction modulate fear relapse and nigrostriatal DA

**Fig. 1:**
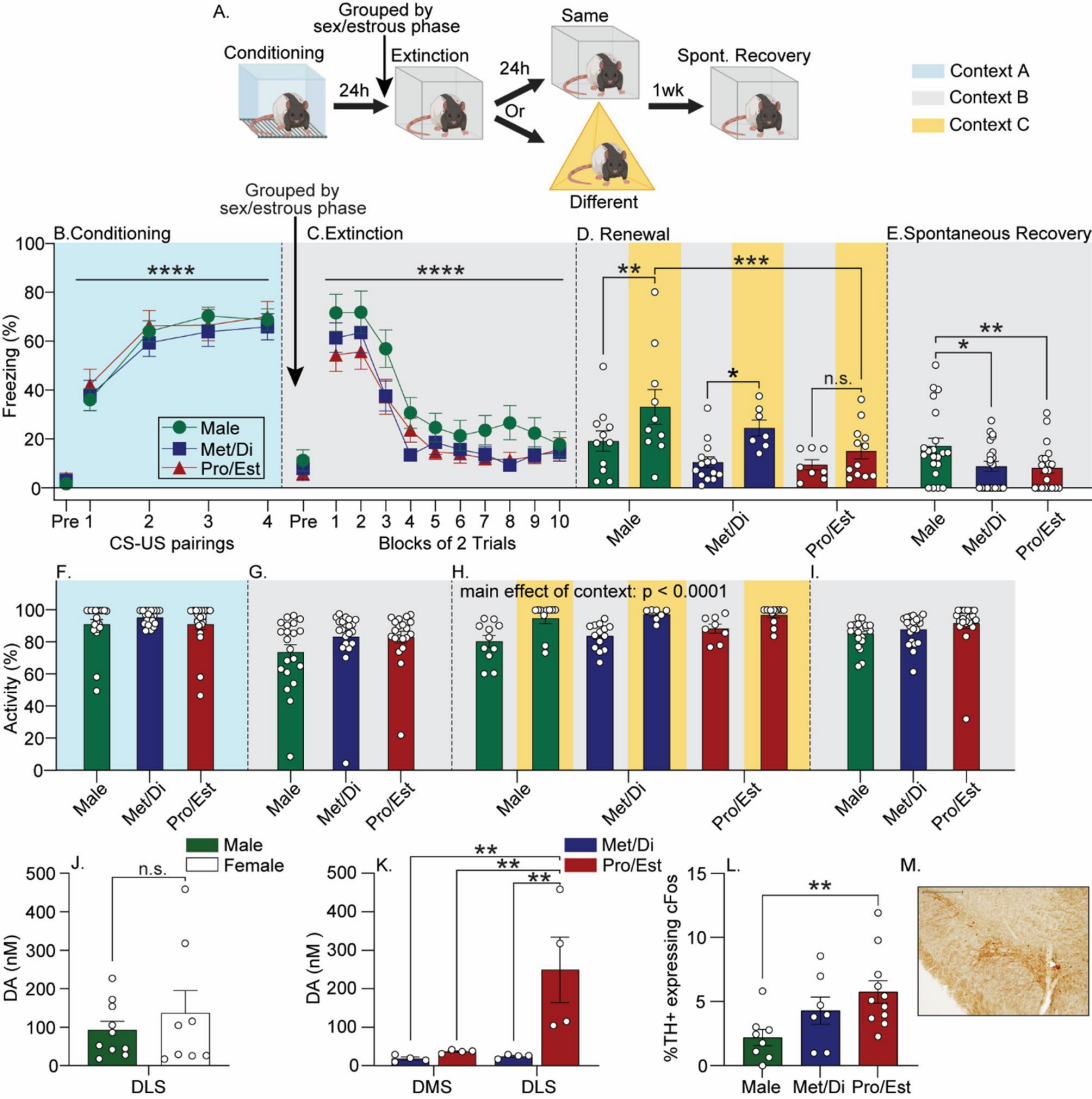
Effects of sex/estrous phase during auditory fear extinction training on fear relapse and nigrostriatal dopamine (DA) **A**) Experimental design. Rats were exposed to auditory fear conditioning in context A (blue). The next day, females in metestrus or diestrus (Met/Di) were grouped together and females in proestrus or estrus (Pro/Est) were grouped together prior to fear extinction training in context B (grey) and 24 h later, re-exposed to the extinguished conditioned stimulus (CS) in either context B, or a novel context C to assess fear renewal. One week later, rats were placed back into context B and re-exposed to the CS to assess spontaneous recovery of fear. **B**-**E**) Levels of freezing during each behavioral session. **F**-**I**) Locomotor activity prior to the first CS during behavioral sessions. **J**) Effect of sex on electrically-evoked DA release in the dorsolateral striatum (DLS). **K**) Effect of female estrous phase on electrically-evoked DA release in the dorsomedial striatum (DMS) and DLS. **L**) Percentage of tyrosine hydroxylase (TH)-positive neurons co-expressing cFos in the substantia nigra (SN) of rats exposed to fear extinction. **M**) Representative photomicrograph depicting double TH+ / cFos + immunoreactivity in the SN. Scale bar = SN 200 μm. All freezing data represent group means ± SEM. *p < 0.05; **p < 0.01; ****p < 0.0001, Bonferroni’s. Abbreviations: US, unconditioned stimulus. Figure was created using BioRender

Here, we assessed the effects of sex and estrous phase during fear extinction on subsequent fear renewal and spontaneous recovery (see Fig. 1A for experimental design). No differences in freezing during any behavioral test were observed between females exposed to fear extinction during Met or Di and Pro or Est; therefore, females were assigned to Met/Di or Pro/Est groups based on estrous phase during fear extinction, as in prior work [10]. After exclusion of rats due to hardware failure (*n* = 3), statistical outliers (*n* = 1), or irregular or ambiguous estrous phases prior to fear extinction (*n* = 6), final group sizes were *n* = 11 (Male/Same), *n* = 10 (Male/Different), *n* = 14 (Met/Di/Same), *n* = 7 (Met/Di/Different), *n* = 8 (Pro/Est/Same), *n* = 12 (Pro/Est/Different).

All rats acquired fear conditioning (F_(3,177)_ = 38.8; *p* < 0.0001; n^2^*p* = 0.4; Fig. 1B), and there were no differences in freezing between males and females subsequently assigned to Pro/Est or Met/Di groups (F_(2,59)_ = 0.5; *p* = 0.6; Fig. 1B). All rats displayed within-session fear extinction (F_(9,531)_ = 68.6; *p* < 0.0001; n^2^*p* = 0.5; Fig. 1C) that was similar between sex/estrous phase groups (F_(2,59)_ = 2.5; *p* = 0.08; Fig. 1C). Due to mixed reports on sex differences in fear renewal [8, 9], renewal was first analyzed by sex. Similar to Schoenberg et al. (2024), both females and males demonstrated fear renewal as indicated by greater freezing in context C than context B, but females froze less than males in both contexts (Supplementary Fig. 1). When females were separated into estrous phase groups, ANOVA revealed significant main effects of sex/estrous phase (F_(2,56 )_ = 6.0; *p* = 0.004; n^2^*p* = 0.2) and context (F_(1, 56)_ = 11.1; *p* < 0.001; n^2^*p* = 0.2) on freezing during renewal testing (Fig. 1D). Post-hoc analyses revealed no group differences in fear extinction memory retention assessed in context B (Fig. 1D). Since averaging the freezing score across extinction trials could obscure potential estrous phase effects on extinction memory retention early in the retention test, freezing in context B was also analyzed across trials with repeated measures ANOVA. No significant sex/estrous phase effects were observed (Supplementary Fig. 2A). Males and Met/Di females displayed fear renewal in context C, while Pro/Est females did not (Fig. 1D). ANOVA revealed no significant main effect of sex/estrous phase on freezing during spontaneous recovery (Supplementary Fig. 3A). However, when freezing in response to the first CS was analyzed separately to assess spontaneous recovery prior to the start of within-session extinction, both Pro/Est and Met/Di females froze less than males (F_(2,59)_ = 3.9; *p* = 0.02; n^2^*p* = 0.1; Fig. 1E). By this point, all but one female had been exposed to fear extinction or extinction memory / renewal sessions during Pro or Est, so it is likely that this sex difference was driven by high levels of ovarian hormones during either of these tests.

There were no sex/estrous phase differences in locomotor activity prior to the first CS during conditioning (F_(2,59)_ = 0.8; *p* = 0.4; Fig. 1F), extinction (F_(2,59)_ = 1.6; *p* = 0.1; Fig. 1G), or spontaneous recovery (F_(2,59)_ = 0.8; *p* = 0.4; Fig. 1I). There was a main effect of context (F_(1,56)_ = 28.7; n^2^*p* = 0.3; *p* < 0.0001; n^2^*p* = 0.3) during the renewal test, but there was no effect of sex/estrous phase (F_(2,56)_ = 1.7; *p* = 0.1; Fig. 1H).

We next evaluated how sex and estrous cycle impact the nigrostriatal DA pathway. No differences between females (*n* = 8) and males (*n* = 10) in stimulus-evoked DA release in the DLS were observed (F_(1,16)_ = 0.5; *p* = 0.4; Fig. 1J). However, when females were broken into estrous phases, we found a main effect of sex/estrous phase (F(_2,15_),  = 6.2, *p* = 0.01; n^2^*p* = 0.1) on stimulated DA release in the DLS. Post-hoc analysis revealed that females in Pro/Est (*n* = 4) had greater DA release compared to males and females in Met/Di (*n* = 4). In females, stimulated DA release was greater in the DLS than the DMS (main effect of region: F_(1,6)_ = 6.5; *p* = 0.04; n^2^*p* = 0.5; estrous phase x region interaction: F_(1,6)_ = 5.8; *p* = 0.05; n^2^*p* = 0.5; Fig. 1K; Supplementary Fig. 4). This pattern of data is similar to that observed with extinction-induced cFos, analysis of which revealed that the %TH + neurons expressing cFos in the substantia nigra of Pro/Est females is not significantly different from Met/Di females but is different from males (F_(2, 23)_ = 4.3, *p* = 0.02; n^2^*p* = 0.3; Fig. 1L and M).

### Estradiol prior to fear extinction reduces fear relapse and increases stimulated DA release in the DLS

**Fig. 2:**
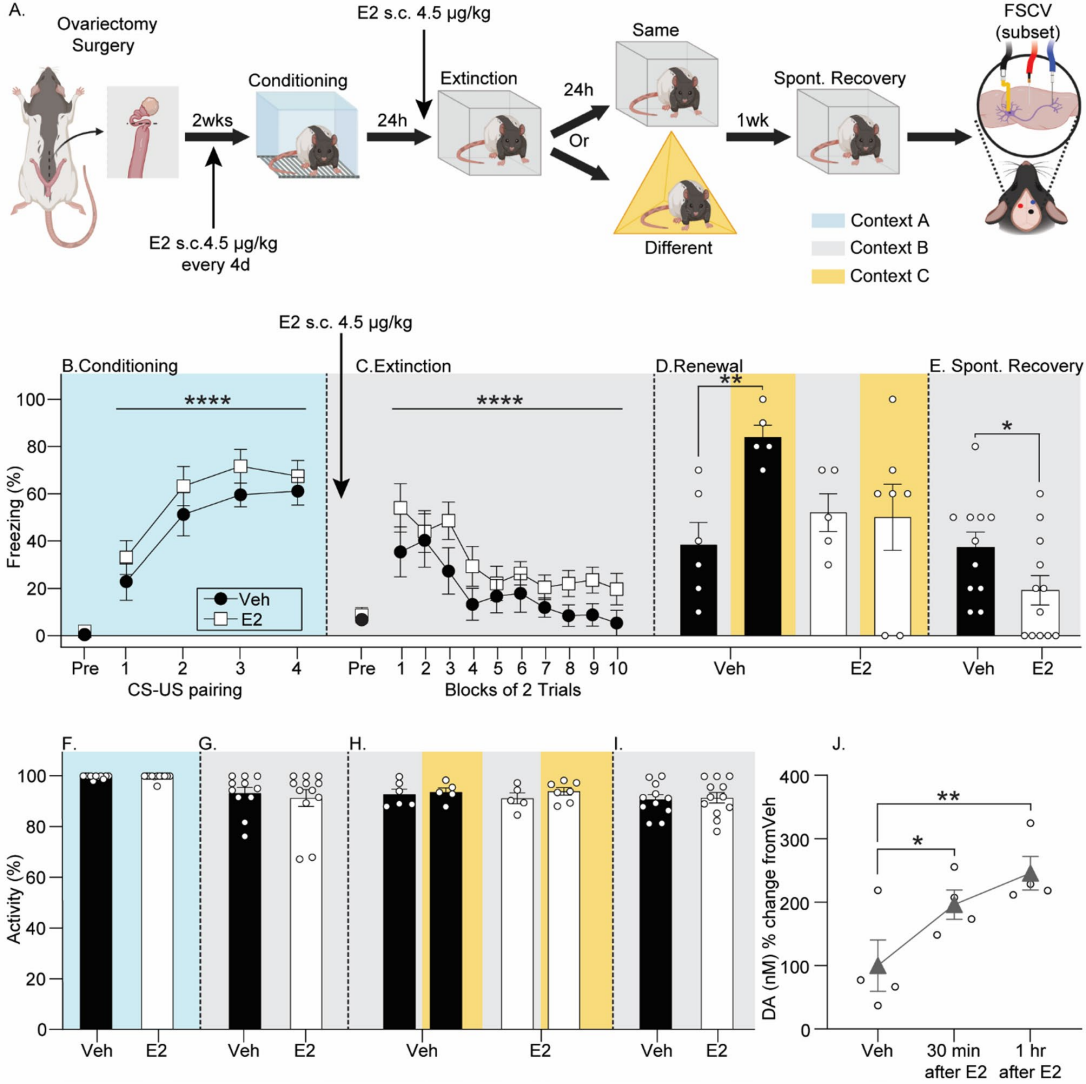
The effects of 17β-estradiol (E2) administration during fear extinction training on fear relapse and dorsolateral striatum (DLS) dopamine (DA). **A**) Experimental design as described in Fig. 1. Behavioral testing began 2 weeks following ovariectomy, during which time all rats received E2 (4.5 mg/kg, s.c.) every 4 days. **B**-**E**) Levels of freezing during behavioral tests. Rats received either vehicle (Veh) or E2 administration 30 min prior to fear extinction training. **F**-**I**) Locomotor activity prior to the first conditioned stimulus (CS) during behavioral sessions. **J**) Electrically-evoked DA release in the DLS after Veh administration, 30 min–1 h after E2 administration. All freezing data represent group means ± SEM. *p < 0.05; **p < 0.01; ****p < 0.0001, Bonferroni’s. Abbreviations: US, unconditioned stimulus; FSCV, fast scan cyclic voltammetry. Figure was created using BioRender

Either progesterone or E2 administered at the time of fear extinction can enhance extinction recall in cycling female rats [39]. However, since the effects of E2 alone on fear extinction and recall in OVX rats are more established than that of progesterone [3, 4, 5, 7], we tested the hypothesis that E2 administration prior to fear extinction reduces fear relapse in OVX females (see Fig. 2A for experimental design). One rat died after surgery, resulting in group sizes of *n* = 6 (Veh/Same), *n* = 5 (Veh/Different), *n* = 5 (E2/Same), *n* = 7 (E2/Different).

All rats acquired auditory fear conditioning (F_(3,63)_ = 14.2, *p* < 0.0001; n^2^*p* = 0.4), and freezing did not differ between drug groups (F_(1,21)_ = 2.5, *p* = 0.1; Fig. 2B). Rats displayed within-session fear extinction (F_(9,189)_ = 10.8, *p* < 0.0001; n^2^*p* = 0.3) that did not differ between drug groups (F_(1,21)_ = 2.4, *p* = 0.1; Fig. 2C). ANOVA revealed a significant main effect of context (F_(1,19)_ = 4.0, *p* = 0.05; n^2^*p* = 0.2) and a significant drug x context interaction (F_(1,19)_ = 4.7, *p* = 0.04; n^2^*p* = 0.2) on freezing during renewal testing (Fig. 2D). E2 failed to enhance fear extinction memory retention assessed by freezing in context B (Fig. 2D; Supplementary Fig. 2B). However, relative to Veh, E2 prior to fear extinction reduced fear renewal (Fig. 2D) and spontaneous recovery (F_(1,21)_ = 4.3, *p* < 0.04; n^2^*p* = 0.2; Fig. 2E).

No group differences in locomotor activity prior to the first CS during conditioning F_(1,21)_ = 0.01, *p* = 0.8; Fig. 2F), extinction (F_(1,21)_ = 0.06, *p* = 0.8; Fig. 2G), renewal (main effect of drug: F_(1,19)_ = 0.01, *p* = 0.9; main effect of context: F_(1,19)_ = 2.0, *p* = 0.1; Fig. 2H), or spontaneous recovery (F_(1,21)_ = 0.5, *p* = 0.8; Fig. 2I) were noted. Compared to Veh, E2 increased stimulus-evoked DA release in the DLS of OVX rats (*n* = 4; F_(2,6)_ = 10.2; *p* = 0.01; n^2^*p* = 0.8; Fig. 2J; Supplementary Fig. 5).

### Inhibition of the SN-DLS pathway during fear extinction restores fear renewal in females exposed to fear extinction during pro/est

**Fig. 3:**
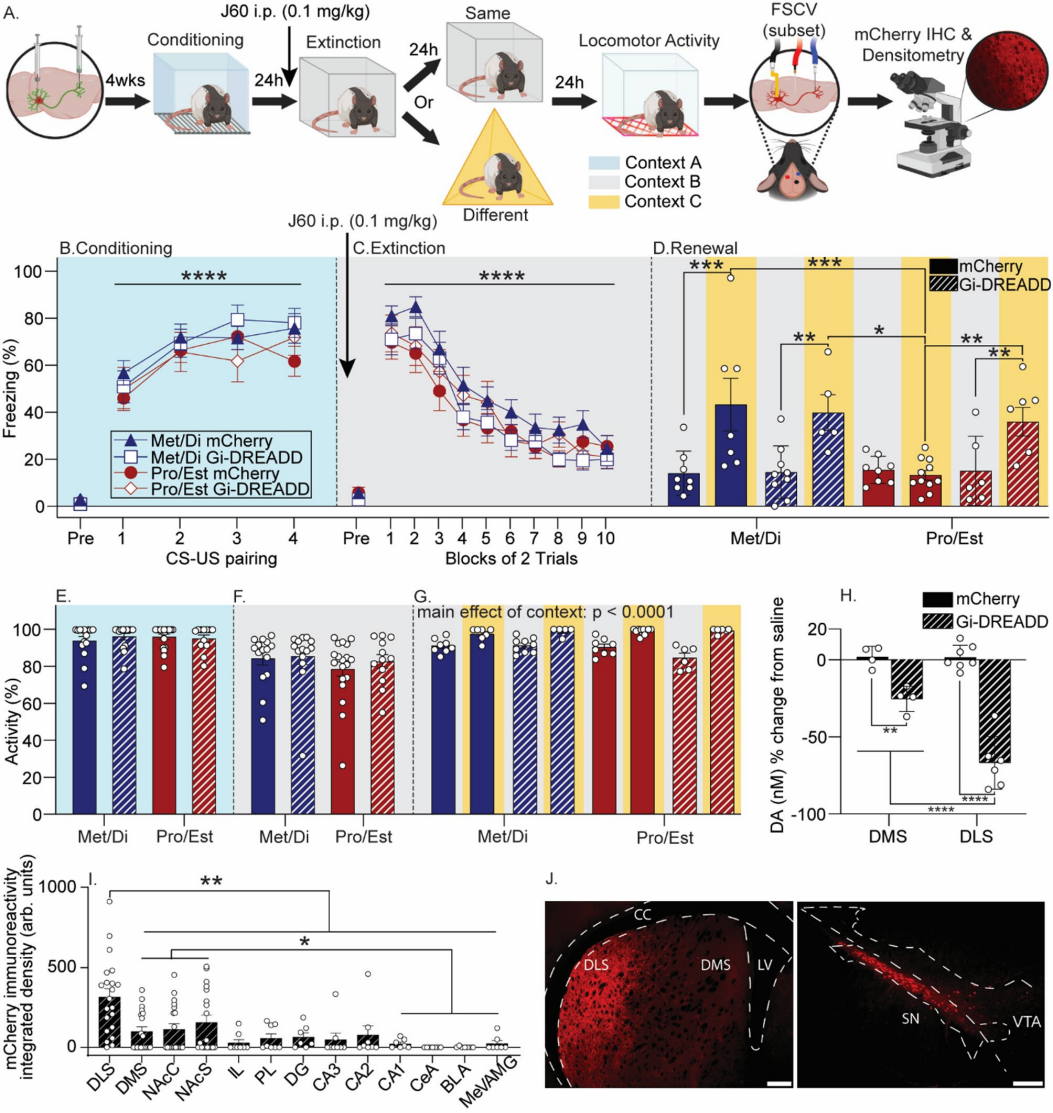
The effects of chemogenetic inhibition of substantia nigra (SN) neurons projecting to the dorsolateral striatum (DLS) during fear extinction on fear renewal in females. **A**) Experimental design as described in Fig. 1. Females received injections of a retrograde adeno-associated virus (AAV) encoding cre-recombinase into the DLS and either AAV-DIO-mCherry (mCherry) or an AAV containing a construct coding for a cre-recombinase-dependent G_i_-coupled designer receptor exclusively activated by designer drug (G_i_-DREADD) into the SN. Behavioral testing began 4 weeks after surgery. **B**-**D**) Levels of freezing during behavioral sessions. All rats received JHU37160 dihydrochloride (J60; 0.1 mg/kg, i.p.) 30 min prior to fear extinction training. **E**-**G**) Locomotor activity prior to the first conditioned stimulus (CS) during behavioral sessions. **H**) Concentration of electrically-evoked dopamine (DA) release in the DLS following J60 administration. **I**) mCherry terminal immunoreactivity in striatal subregions and other brain regions implicated in fear extinction in rats expressing G_i_-DREADD. **J**) Representative photomicrographs depicting mCherry in a rat injected with G_i_-DREADD. Scale bar = DLS 200 μm; SN 100 μm. All freezing data represent group means ± SEM. *p < 0.05; **p < 0.01; ***p < 0.001; ****p < 0.0001, Bonferroni’s. Abbreviations: US, unconditioned stimulus; FSCV, fast scan cyclic voltammetry; IHC, immunohistochemistry; DMS, dorsomedial striatum; NAcC, nucleus accumbens core; NAcS, nucleus accumbens shell; IL, infralimbic cortex; PL, prelimbic cortex; DG, dentate gyrus; CA3, cornu ammonis 3, CA2, cornu ammonis 2, CA1, cornu ammonis 1; CeA, central amygdala; BLA, basolateral amygdala; mAMG, medial amygdala; CC, corpus callosum; LV, lateral ventricle. Figure was created using BioRender

Both stimulation of SN DA neurons [27] and increasing D1R signaling in the DLS [28] during fear extinction reduces fear renewal in males, raising the possibility that potentiated DA release in the DLS observed during Pro/Est contributes to the acquisition of relapse-resistant fear extinction. Here, intersectional chemogenetics was used to inhibit the SN-DLS pathway during fear extinction in Pro/Est and Met/Di females and subsequent fear renewal was assessed (see Fig. 3A for experimental design). After exclusion of rats with nebulous estrous cycles (*n* = 2), fatalities from surgery (*n* = 7), and missed viral injection (*n* = 5), final group sizes were *n* = 8 (Met/Di/mCherry/Same), *n* = 7 (Met/Di/mCherry/Different), *n* = 9 (Met/Di/G_i_-DREADD/Same), *n* = 5 (Met/Di/G_i_-DREADD/Different), *n* = 8 (Pro/Est/mCherry/Same), *n* = 11 (Pro/Est/mCherry/Different), *n* = 6 (Pro/Est/G_i_-DREADD/Same), *n* = 6 (Pro/Est/G_i_-DREADD/Different).

All rats acquired auditory fear conditioning (F_(3,168)_ = 21.0, *p* < 0.0001; n^2^*p* = 0.3; Fig. 3B) that did not differ between virus (F_(1,56)_ = 0.01, *p* = 0.9; Fig. 3B) or subsequently assigned extinction estrous phase groups (F_(1,56)_ = 2.0, *p* = 0.1; Fig. 3B). Rats displayed within-session fear extinction (F_(9,504)_ = 84.0, *p* < 0.0001; n^2^*p* = 0.6; Fig. 3C) that did not differ between virus groups (F_(1,56)_ = 0.4, *p* = 0.5; Fig. 3C) or estrous phase (F_(1,56)_ = 0.8, *p* = 0.3; Fig. 3C). ANOVA revealed significant main effects of estrous phase (F_(1,52)_ = 4.3, *p* = 0.04; n^2^*p* = 0.08) and context (F_(1,52)_ = 22.6, *p* < 0.0001; n^2^*p* = 0.3), as well as a significant estrous phase x context interaction (F_(1,52)_ = 5.4, *p* = 0.02; n^2^*p* = 0.09) on freezing during the renewal test (Fig. 3D). Again, there were no group differences in fear extinction retention in context B (Fig. 3D; Supplementary Fig. 2C). Post-hoc analyses revealed typical fear renewal in Met/Di, but not Pro/Est females expressing mCherry (Fig. 3D). Inhibition of the SN-DLS pathway during fear extinction had no effect on fear renewal in Met/Di females, but restored fear renewal in Pro/Est females (Fig. 3D).

There were no group differences in locomotor activity prior to the first CS during conditioning (main effect of virus: F_(1,56)_ = 0.1, *p* = 0.6; main effect of estrous phase: F_(1,56)_ = 0.01, *p* = 0.9; Fig. 3E) or extinction (main effect of virus: F_(1,56)_ = 0.9, *p* = 0.3; main effect of estrous phase: F_(1,56)_ = 0.4, *p* = 0.4; Fig. 3F). There was a main effect of context (F_(1,52)_ = 91.7, *p* < 0.0001; n^2^*p* = 0.6; Fig. 3G) during the renewal test, but there was no effect of viral treatment (F_(1,52)_ = 1.2, *p* = 0.2) or estrous phase (F_(1,52)_ = 2.2, *p* = 0.1). SN-DLS pathway inhibition did not impact locomotor activity in locomotor activity chambers (Supplementary Fig. 6A).

FSCV was used to assess the ability of G_i_-DREADD to suppress DA release in the DLS and DMS in a subset of rats exposed to behavioral testing and expressing mCherry (*n* = 11) or G_i_-DREADD (*n* = 10). We did not include sufficient numbers of Pro/Est and Met/Di rats in each group to analyze FSCV data by estrous phase during extinction. However, we found no effect of estrous phase at the time of FSCV on the % DA suppression caused by J60 in Gi-DREADD rats (F_(1,13)_ = 0.9, *p* = 0.3), so rats in various estrous phases were combined. J60 suppressed electrically-evoked DA release in both the DLS and DMS of rats that received G_i_-DREADD, relative to rats that received mCherry (main effect of virus: F_(1,17)_ = 88.4, *p* < 0.0001; n^2^*p* = 0.8; Fig. 3H; Supplementary Fig. 7). J60 elicited a larger suppression of DA release in the DLS than the DMS (main effect of brain region: F_(1,17)_ = 16.7, *p* = 0.0008; n^2^*p* = 0.4; virus x brain region interaction: F_(1,17)_ = 16.3, *p* = 0.0009; n^2^*p* = 0.5; Fig. 3E; Supplementary Fig. 7). DLS-projecting SN neurons branch and project to other regions [40]; therefore, terminal mCherry immunoreactivity was quantified in regions implicated in fear extinction with densitometry in rats injected with hM4Di. mCherry expression did not vary by estrous phase at the time of extinction (F_(1,13)_ = 0.07, *p* = 0.78) or euthanasia (F_(1,13)_ = 0.004, *p* = 0.94), so rats were combined into one group. Terminal mCherry expression differed by brain region, with the highest expression in the DLS (F_(13,145)_ = 4.8; *p* < 0.0001; n^2^*p* = 0.3; Fig. 3I and J).

### Stimulation of the SN-DLS pathway during fear extinction reduces fear renewal in males

**Fig. 4:**
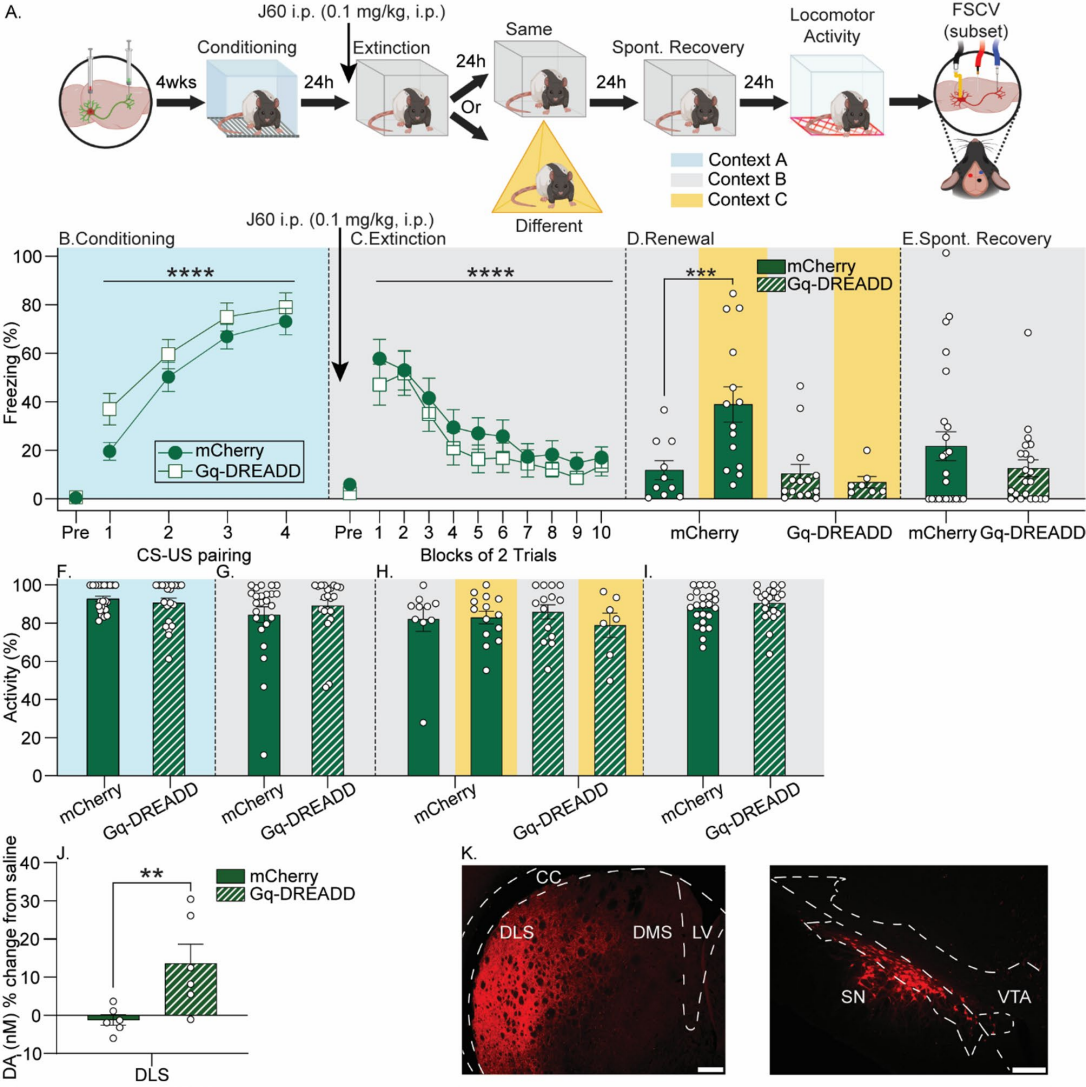
The effects of chemogenetic activation of substantia nigra (SN) neurons projecting to the dorsolateral striatum (DLS) during fear extinction on fear relapse in males. **A**) Experimental design as described in Fig. 1. Male rats received injections of a retrograde adeno-associated virus (AAV) encoding cre-recombinase into the DLS and either AAV-DIO-mCherry (mCherry) or an AAV containing a construct coding for a cre-recombinase-dependent G_q_-coupled designer receptor exclusively activated by designer drug (G_q_-DREADD) into the SN. Behavioral testing began 4 weeks after surgery. **B**-**E**) Levels of freezing during behavioral sessions. All rats received JHU37160 dihydrochloride (J60) 30 min prior to fear extinction training. **F**-**I**) Locomotor activity prior to the first conditioned stimulus (CS) during behavioral sessions. **J**) Concentration of electrically-evoked dopamine (DA) release in the DLS following J60 administration. **K**) Representative photomicrographs depicting mCherry in rats injected with G_q_-DREADD. Scale bar = DLS 200 μm; SN 100 μm. All freezing data represent group means ± SEM. **p < 0.01; ***p < 0.001; ****p < 0.0001, Bonferroni’s. Abbreviations: US, unconditioned stimulus; FSCV, fast scan cyclic voltammetry; CC, corpus callosum; LV, lateral ventricle. Figure was created using BioRender

Here we used excitatory intersectional chemogenetics to test the hypothesis that SN-DLS pathway stimulation during fear extinction reduces fear relapse in males (experimental design shown in Fig. 4A). After exclusion of fatalities from surgery (*n* = 5), statistical outliers (*n* = 4), and missed viral injections (*n* = 6), final group sizes were *n* = 10 (mCherry/Same), *n* = 14 (mCherry/Different), *n* = 14 (G_q_-DREADD/Same), *n* = 7 (G_q_-DREADD/Different).

All rats acquired auditory fear conditioning (F_(3,129)_ = 52.4, *p* < 0.0001; n^2^*p* = 0.5) that did not differ between virus groups (F_(1,43)_ = 2.7, *p* = 0.1; Fig. 4B). Rats displayed within-session fear extinction (F_(9,387)_ = 25.3, *p* < 0.0001; n^2^*p* = 0.4) that did not differ between viral groups (F_(1,43)_ = 0.8, *p* = 0.3; Fig. 4C). ANOVA revealed a significant main effect of virus (F_(1,41)_ = 8.5, *p* = 0.005; n^2^*p* = 0.2), context (F_(1,41)_ = 4.2, *p* = 0.04; n^2^*p* = 0.09) and context x virus interaction (F_(1,41)_ = 7.2, *p* = 0.01; n^2^*p* = 0.1) on freezing during renewal (Fig. 4D). No effect of SN-DLS pathway stimulation during fear extinction on extinction memory retention assessed by freezing in context B was found (Fig. 4D; Supplementary Fig. 2D). Post-hoc analyses revealed that rats expressing mCherry displayed typical fear renewal, but G_q_-DREADD rats did not (Fig. 4D). Stimulation of the SN-DLS pathway during fear extinction did not impact spontaneous recovery when freezing was averaged across trials (Supplementary Fig. 3B) or during the first trial (F_(1,43)_ = 1.6, *p* = 0.2; Fig. 4E).

There were no group differences in locomotor activity prior to the first CS during conditioning (F_(1,43)_ = 0.5, *p* = 0.4; Fig. 4F), extinction (F_(1,43)_ = 0.7, *p* = 0.3; Fig. 4G), renewal (main effect of virus: F_(1,41)_ = 0.1, *p* = 0.7; main effect of context: F_(1,41)_ = 1.0, *p* = 0.3;Figure 4H), or spontaneous recovery (F_(1,43)_ = 0.6, *p* = 0.4; Fig. 4I). No effect of SN-DLS pathway stimulation on locomotor activity in locomotor activity chambers was noted (Supplementary Fig. 6B). J60 potentiated electrically-evoked DA release in the DLS of G_q_-DREADD rats (*n* = 6) compared to mCherry (*n* = 6; F_(1,10)_ = 8.2, *p* = 0.01; n^2^*p* = 0.4; Fig. 4J; Supplementary Fig. 8). Viral mCherry expressed robustly in the SN-DLS pathway (Fig. 4K).

## Discussion

Here we report the novel finding that estrous phase during fear extinction modulates later fear relapse through a mechanism involving nigrostriatal DA. Females in Pro/Est during fear extinction had less fear renewal and spontaneous recovery than males and females in Met/Di during extinction. E2 seems to be the ovarian hormone important for mediating the observed effects of estrous cycle, as E2 administration in OVX females mimicked the effects of Pro/Est. Relative to males, females in Pro/Est had greater activity of SN DA neurons during extinction, and both Pro/Est and E2 administration potentiated electrically-evoked DA release in the DLS. DLS DA is critical for ovarian hormone modulation of fear extinction and relapse, since SN-DLS pathway inhibition during fear extinction restored fear renewal in Pro/Est females and stimulating the SN-DLS pathway during extinction reduced fear renewal in males. These results provide new insight into how ovarian hormones modulate fear extinction in a relapse-resistant manner.

Current results could help clarify discrepancies in the literature regarding sex differences in fear renewal. Similar to Schoenberg et al. (2024), we observed fear renewal in both males and females, although females froze less than males (Supplementary Fig. 1). These results contrast with those of Binette et al. (2022), who report a lack of fear renewal in females. Discrepancies between studies could be due to differences in the strain of rats used, as Long Evans rats were used here and in Schoenberg et al. (2024), but Binette et al. (2022) used a transgenic line of Wistar rats. Since estrous phase during fear extinction influences later renewal, differences between studies could also be explained by variations in arbitrary alignment of fear extinction training sessions to estrous cycle phases, which were not measured in either prior study. Importantly, in addition to impacting fear renewal, we found that high levels of ovarian hormones, either through natural fluctuations during extinction or renewal testing (which can serve as an additional extinction session) or E2 administration prior to extinction, also impact spontaneous recovery. This observation suggests that high levels of ovarian hormones during fear extinction could broadly reduce multiple forms of relapse in females, an observation with potential clinical implications.

The consensus in the literature is that high levels of ovarian hormones, particularly E2, at the time of fear extinction promotes the retention of fear extinction, indicated by a reduction in freezing during extinction memory testing [3, 4, 5, 6]. Notably, this effect was not observed here (Supplementary Fig. 2) or in our prior work [10]. One explanation for this discrepancy could be differences in the strength of the fear conditioning memory between studies. We specifically use conditioning and extinction conditions that elicit fear renewal. This necessitates low levels of freezing in the “same” context, which could obscure potential group differences in fear extinction retention in this context. Indeed, most of the prior work reporting that estrous cycle at the time of fear extinction impacts extinction retention used a greater number of CS-US pairings during conditioning than we use in the current studies.

Despite the failure to observe enhanced fear extinction retention in rats exposed to extinction during conditions of high ovarian hormones, we nonetheless observed less fear relapse in these rats. If improved extinction retention in high hormone groups (i.e., Pro/Est, E2) was obscured by a floor effect in the current studies, the reduction in relapse could nevertheless be a product of improved fear extinction retention. Alternatively, ovarian hormones during fear extinction could impact fear relapse through a mechanism independent of enhanced fear extinction memory retention, per se. Supporting this possibility, the reduced fear renewal in female, compared to male, rats reported by Binette et al. (2022) similarly occurred in the absence of improved fear extinction retention in females. Moreover, both stimulation of the SN-DLS pathway (Fig. 4D) and increasing D1R signaling in the DLS during fear extinction [28] reduce fear renewal in the absence of an observed effect on extinction memory retention. Since high levels of ovarian hormones enhance SN DA neural activity during fear extinction and potentiate DA release in the DLS, it is possible that ovarian hormones could recruit a mechanism involving DA in the DLS that renders fear extinction memory resistant to relapse independently from the strength of the fear extinction memory. Future research will be required to understand how nigrostriatal DA impacts fear relapse independently of fear extinction.

We observed that the estrous cycle impacts the nigrostriatal DA pathway at both the cell body and terminal regions. Although the percentage of SN DA neurons expressing cFos after fear extinction was very low, it was nonetheless greatest in females exposed to fear extinction during Pro/Est (Fig. 1L). Ovarian hormones are thought to modulate DA transmission through DA terminal disinhibition rather than acting directly at the cell body [41, 42]. Thus, greater extinction-induced SN DA activity observed during Pro/Est could be mediated by feedback loops through the SN reticularis [40]. Within the DS, estrous phase had a more pronounced effect on DA release in the DLS than the DMS, indicating that ovarian hormones can influence DA transmission differently in different DS subregions. Together, the data support the possibility that high levels of ovarian hormones during fear extinction could reduce relapse by potentiating DA release in the DLS, but they do not completely rule out a potential contribution from DA in other striatal regions.

The observation that inhibition of the SN-DLS pathway during fear extinction did not impact fear extinction acquisition, retention, or renewal in Met/Di females (Fig. 3D) is consistent with our recent observation that temporary inhibition of the DLS during fear extinction has no impact on fear extinction or renewal in male rats [28]. These data suggest that the SN-DLS pathway may not contribute to “normal” fear extinction or relapse. However, SN-DLS pathway inhibition during fear extinction restored renewal in females exposed to fear extinction during Pro/Est. Thus, when it is recruited during fear extinction by the presence of ovarian hormones, heightened activity of the SN-DLS pathway renders fear extinction memory resistant to relapse. In fact, stimulation of the SN-DLS pathway during fear extinction seems to be sufficient to reduce renewal in the absence of high levels of ovarian hormones, as stimulation of the SN-DLS pathway (Fig. 4D) or a D1R agonist injected into the DLS [28] during fear extinction both reduce renewal in males. These data suggest that targeting SN-DLS pathway activity during fear extinction could be a means to reduce relapse in both sexes. Future studies should stimulate the SN-DLS pathway during extinction in Met/Di females to clarify the role of this pathway in estrous cycle-modulation of fear relapse.

We assessed the anatomical specificity of the intersectional approach to target the SN-DLS pathway. The highest levels of terminal mCherry were observed in the DLS, consistent with the large suppression of electrically-evoked DA caused by J60 in rats expressing G_i_-DREADDs. However, some mCherry was observed in other striatal regions, and J60 also suppressed DA release in the DMS, albeit to a lesser degree than the DLS (Fig. 3H). The possibility that alterations in DA transmission in other striatal regions contributes to the effects of chemogenetic manipulations, therefore, cannot be entirely ruled out. These data have important implications for other studies utilizing intersectional chemogenetics to target “projection-defined” DA pathways.

Interestingly, the current results add to growing evidence suggesting that females, compared to males, preferentially recruit DLS circuits to guide behavior. Formation of habitual responding during operant training typically involves a shift from DMS- to DLS-control of operant behavior [43, 44], and females form habits earlier during operant training than do males [45, 46]. Female rats given the opportunity to press a lever to escape from electric shock do so using the DLS, whereas males use the DMS to accomplish the identical instrumental task [47]. When provided with a running wheel in their cages, both sexes of rats will engage in robust wheel running behavior. However, females have a preference to use the DLS to govern the acquisition of wheel running, whereas males prefer the DMS [48]. Here, we find that females also recruit DLS circuits during fear extinction, a task not typically associated with the DS, and that this is influenced by ovarian hormones.

## Perspectives and significance

The presence of ovarian hormones during fear extinction reduces the return of fear in rodents, highlighting the importance of considering sex and estrous phase in fear extinction experiments and when considering factors that could influence the outcome of exposure therapy in human patients. The fact that reductions in fear relapse caused by high levels of ovarian hormones or SN-DLS pathway stimulation during extinction were observed in the absence of improved fear extinction retention suggests that strengthening of extinction memory is not necessarily a prerequisite for the effectiveness of potential therapeutic manipulations to reduce fear relapse. Therefore, focusing on fear relapse rather than fear extinction retention per se may increase the translational potential of pre-clinical work on fear extinction. Further research is essential to identify how ovarian hormone-mediated recruitment of the SN-DLS DA pathway interacts with canonical fear extinction circuitry to render fear extinction memory resistant to relapse.

## Conclusions

In summary, high levels of ovarian hormones present during fear extinction render fear extinction resistant to relapse through a nigrostriatal pathway. Future work is required to understand how SN-DLS pathway activity during extinction reduces later relapse. Regardless of the mechanism, the SN-DLS pathway should be considered a potential target for the reduction of fear relapse after extinction in both sexes.

## Electronic supplementary material

Below is the link to the electronic supplementary material.

Supplementary Material 1

## Data Availability

Data are available upon reasonable request.
