# Supplementary material for: High ovarian hormones present during fear extinction reduce fear relapse through a nigrostriatal dopamine pathway
Source: Biol Sex Differ. 2025 Jun 1;16:38. doi: 10.1186/s13293-025-00722-7 (PMC12128558; doi:10.1186/s13293-025-00722-7)
Supplement: Supplementary file 1 — Supplementary Material 1 [file 13293_2025_722_MOESM1_ESM.docx]

**Supplementary Figures**


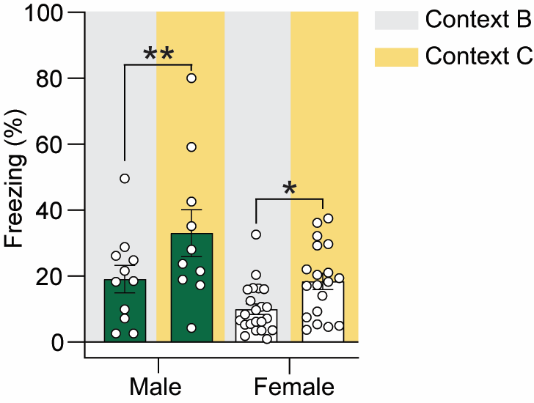


Supplementary Figure 1. Effects of sex on fear renewal. Given discrepancy in the literature regarding sex differences in fear renewal, freezing during the renewal test was analyzed between sexes with analysis of variance. Rats displayed greater freezing in context C than context B (F_(1,58)_ = 10.4, p < 0.01; n^2^p = 0.1) and females froze less than males (F_(1,58)_ = 11.7, p < 0.001; n^2^p = 0.2). All data represent groups means ± SEM. *p < 0.05; **p < 0.01, Bonferroni’s.


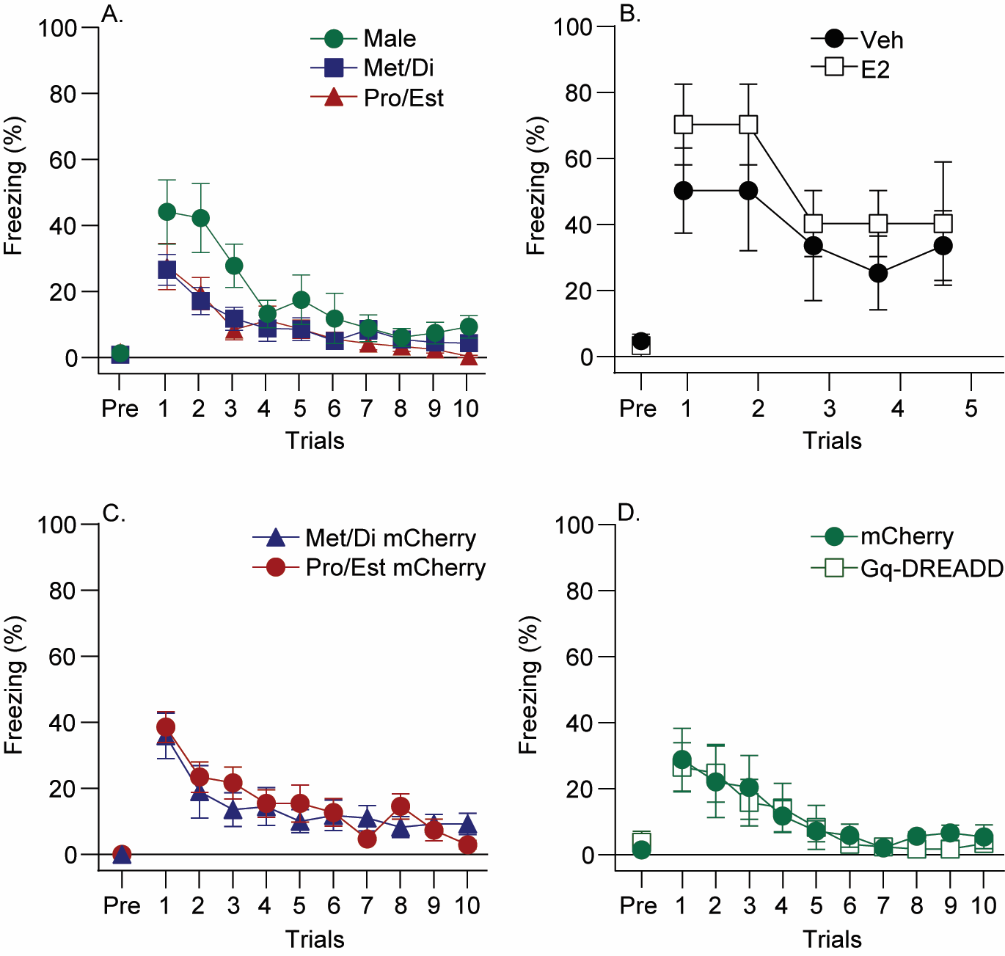


Supplementary Figure 2. Extinction memory retention in context B. Prior studies report that female rodents exposed to fear extinction training during conditions of high 17β-estradiol (E2) have improved extinction retention compared to low E2 females (1-4). Since averaging the freezing observed in the fear extinction context on the day of fear renewal testing could obscure potential group differences in fear extinction retention occurring early in the retention test, we analyzed freezing across time in rats placed back into the fear extinction context on the day of fear renewal testing to determine if manipulations during fear extinction in these studies influences fear extinction retention. A) Repeated measures analysis of variance revealed a trend for a main effect of sex/estrous phase (F_(2,30)_ = 3.0, p = 0.06). B) Administration of E2 (4.5 μg/kg, s.c.) prior to fear extinction had no impact on fear extinction retention relative to vehicle (Veh; 0.1mL/kg, s.c.; F_(1,9)_ = 1.1, p = 0.3). C) Female rats received adeno-associated virus (AAV) encoding cre-recombinase into the dorsolateral striatum (DLS) and AAV-DIO-mCherry (mCherry) into the substantia nigra (SN) and received J60 (0.1 mg/kg, i.p.) 30 minutes prior to fear extinction. No differences in fear extinction retention were observed between rats exposed to fear extinction during metestrus or diestrus (Met/Di) compared to those in proestrus or estrus (Pro/Est; F_(1,14)_ = 0.1, p = 0.7). D) Male rats received AAV encoding cre-recombinase into the DLS and either pAAV8-hSyn-DIO-mCherry (mCherry) or pAAV8-hSyn-DIO-hM3D(G_q_)-mCherry (G_q_-DREADD) into the SN. J60 30 minutes prior to fear extinction had no impact on fear extinction retention in Gq-DREADD rats compared to mCherry (F_(1, 22)_ = 0.06, p = 0.8).


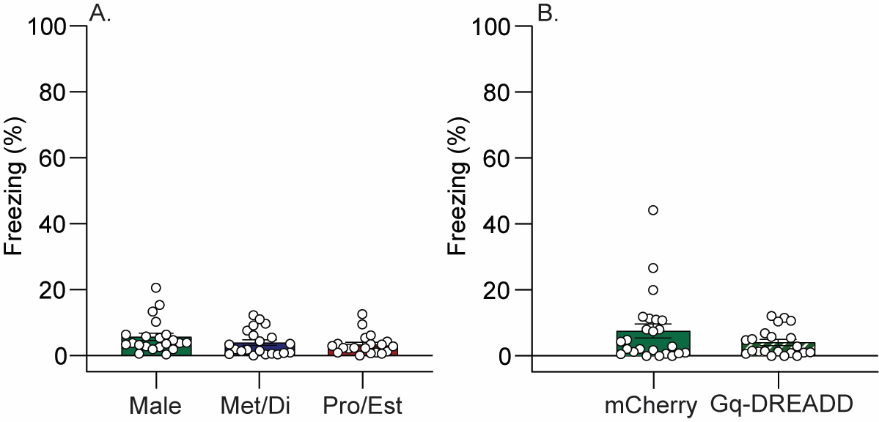


Supplementary Figure 3. Average freezing during all spontaneous recovery trials. A) There were no differences in freezing during spontaneous recovery between males and females in metestrus or diestrus (Met/Di) or proestrus and estrus (Pro/Est; F_(2,59)_ = 1.8, p = 0.1). B) Male rats received adeno-associated virus (AAV) encoding cre-recombinase into the DLS and either pAAV8-hSyn-DIO-mCherry (mCherry) or pAAV8-hSyn-DIO-hM3D(G_q_)-mCherry (G_q_-DREADD) into the SN and received J60 30 minutes prior to fear extinction. No differences in average freezing during spontaneous recovery were observed between mCherry and Gq-DREADD (F_(1,43)_ = 2.1, p = 0.1).


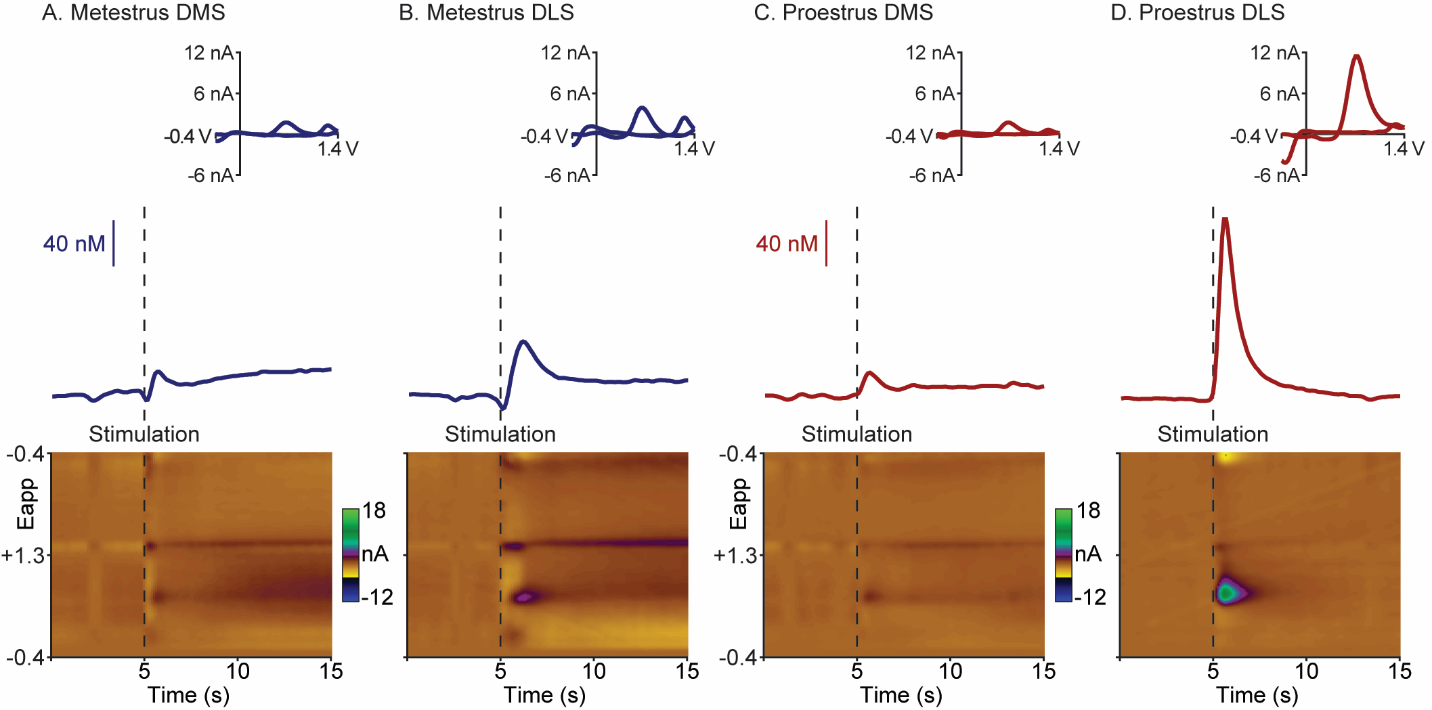


Supplementary Figure 4. Electrically-evoked dopamine (DA) release in dorsal striatal subregions during different phases of the estrous cycle (see Figure 1). A) Representative examples are shown of measurements taken in the dorsomedial striatum (DMS) and B) dorsolateral striatum (DLS) of a single rat during the Metestrus (Met) phase of the estrous cycle, as well as the C) DMS and D) DLS of a single rat during Proestrus (Pro). A, B, C and D) The effect of electrical stimulation on DA release is shown as color plots (Bottom) and corresponding concentration traces (Middle) with cyclic voltammograms (Top). Each set of plots represents data collected during a single recording. Color plots depict time (seconds; x-axis), scan potential applied to the electrode (Eapp [V]; y-axis), and voltammetric current (nA; z-axis). Electrical stimulation (24 pulses, 60 Hz, 300 μA, 2 ms/phase, biphasic) of the substantia nigra is indicated by the black dashed lines. Following stimulation, a DA release event is observed in color plots at the oxidative potential for DA (+0.6 V). The corresponding concentration traces illustrate the concentration of DA (nM; y-axis) across time (seconds; x-axis). Concentration (nM) was converted from current (nA) using a calibration factor. Cyclic voltammograms represent the peak concentration seen in the concentration traces following electrical stimulation and verify the signal as DA. Cyclic voltammograms are plotted as current (nA; y-axis) as a function of applied potential (V; x-axis).

Supplementary Figure 5. Effect of 17β-estradiol (E2) administration on electrically-evoked dopamine (DA) release in the dorsolateral striatum (DLS) in ovariectomized female rats (see Figure 2). Recordings were taken 30 minutes after vehicle (Veh; 0.1 mL/kg, s.c.) administration to establish a baseline response to electrical stimulation. E2 (4.5 μg/kg, s.c.) was then administered, and recordings were taken 30 minutes and 1 hour after injection. A) Representative examples are shown of DA measured in the DLS of a single rat 30 minutes after Veh injection, B) 30 minutes after E2, and C) 1 hour after E2. During each recording, the substantia nigra was electrically stimulated (24 pulses, 60 Hz, 300 μA, 2 ms/phase, biphasic) after 5 seconds and subsequent DA release events were recorded. A-C) The effect of electrical stimulation on DA release is shown as color plots (Bottom) and corresponding concentration traces (Middle) with cyclic voltammograms (Top). Each set of plots represents data collected during a single recording. Stimulation is indicated in the color plots and concentration traces by the black dashed lines. See Supplementary Figure 4 for a description of the representative voltammetric plots.


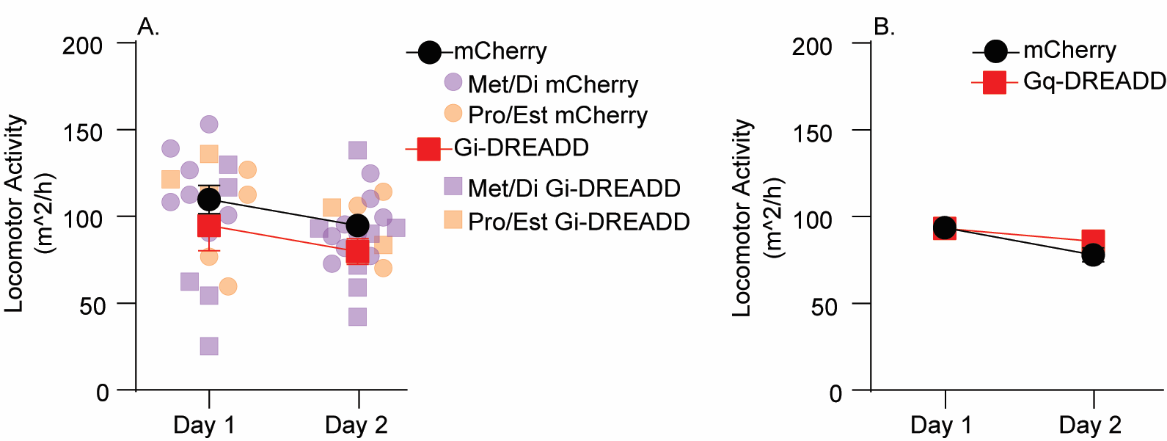


Supplementary Figure 6. Effects of chemogenetic manipulations on locomotor activity. A) Female rats expressing retrograde adeno-associated virus (AAV) encoding cre-recombinase into the dorsolateral striatum (DLS) and either AAV-DIO-mCherry (mCherry) or an AAV containing a construct coding for a cre-recombinase-dependent G_i_-coupled designer receptor exclusively activated by designer drug (G_i_-DREADD) into the substantia nigra were placed into Med Associates locomotor activity chambers for 1 hour per day, for 2 consecutive days. DREADD ligand JHU37160 dihydrochloride (J60, 0.1 mg/kg, i.p.) was administered 30 minutes prior to placement into locomotor activity chambers on day 1 only. Estrous phases (metestrus or diestrus (Met/Di); proestrus or estrus (Pro/Est) were identified with vaginal lavage prior to behavioral testing on both days. There was a main effect of time (F_(1,17)_ = 4.7; p < 0.05; n^2^p = 0.2), but no difference between mCherry or G_i_-DREADD was observed (F_(1,17)_ = 1.9; p = 0.1). Individual data points represent individual subjects in either metestrus or diestrus (Met/Di) or proestrus or estrus (Pro/Est). B) Male rats expressing mCherry or G_q_-DREADD were placed into Med Associates locomotor activity chambers for 1 hour per day, for 2 consecutive days. J60 was administered 30 minutes prior to placement into locomotor activity chambers on day 1 only. There was a main effect of time (F_(1,43)_ = 14.2; p < 0.001; n^2^p = 0.2), but no difference between mCherry or G_q_-DREADD was observed (F_(1,43)_ = 0.3; p = 0.6).

Supplementary Figure 7. Effect of JHU37160 dihydrochloride (J60) administration on electrically-evoked dopamine (DA) release in dorsal striatal subregions of rats injected with retrograde AAV encoding cre-recombinase into the dorsolateral striatum (DLS) and either AAV-DIO-mCherry (mCherry) or an adeno-associated virus (AAV) containing a construct coding for a cre-recombinase-dependent G_i_-coupled designer receptor exclusively activated by designer drug (G_i_-DREADD) into the substantia nigra (SN; see Figure 3). A) Representative fast scan cyclic voltammetry data are shown of recordings taken in the DLS of a single mCherry rat 30 minutes after saline injection and B) 30 minutes after J60 injection, as well as C) recordings taken in the DLS of a single G_i_-DREADD virus rat 30 minutes after saline injection, and D) 30 minutes after J60 injection. Recordings were taken in the dorsomedial striatum (DMS) to determine the specificity of the intersectional approach used to target the SN-DLS pathway. E) Representative examples are shown of recordings taken in the dorsomedial striatum (DMS) of a single mCherry rat 30 minutes after saline and F) 30 minutes after J60 F), as well as G) recordings taken in the DMS of a single G_i_-DREADD virus rat 30 minutes after saline and H) 30 minutes after J60. During each recording, the SN was electrically stimulated (24 pulses, 60 Hz, 300 μA, 2 ms/phase, biphasic) after 5 seconds and subsequent DA release events were recorded. A-H) The effect of electrical stimulation on DA release is shown as color plots (Bottom) and corresponding concentration traces (Middle) with cyclic voltammograms (Top). Each set of plots represents data collected during a single recording. Stimulation is indicated in the color plots and concentration traces by the black dashed lines. See Supplementary Figure 4 for a description of the representative voltammetric plots.

Supplementary Figure 8. Effect of JHU37160 dihydrochloride (J60) administration on electrically-evoked dopamine (DA) release in the dorsolateral striatum (DLS) of rats injected with retrograde adeno-associated virus (AAV) encoding cre-recombinase into the DLS and either AAV-DIO-mCherry (mCherry) or AAV containing a construct coding for a cre-recombinase-dependent G_q_-coupled designer receptor exclusively activated by designer drug (G_q_-DREADD) into the substantia nigra (SN; see Figure 4). Recordings were taken 30 minutes after saline (1 mL/kg, i.p.) injection to establish a baseline DA response to electrical stimulation. J60 (0.1 mg/kg, i.p.) was then given, and recordings were taken 30 minutes after injection. A) Representative fast scan cyclic voltammetry data are shown of recordings taken in the DLS of a single mCherry rat 30 minutes after saline injection and B) 30 minutes after J60 injection, as well as C) recordings taken in the DLS of a single G_q_-DREADD virus rat 30 minutes after saline injection and D) 30 minutes after J60 injection. During each recording, the SN was electrically stimulated (24 pulses, 60 Hz, 300 μA, 2 ms/phase, biphasic) after 5 seconds and subsequent DA release events were recorded. A-D) The effect of electrical stimulation on DA release is shown as color plots (Bottom) and corresponding concentration traces (Middle) with cyclic voltammograms (Top). Each set of plots represents data collected during a single recording. Stimulation is indicated in the color plots and concentration traces by the black dashed lines. See Supplementary Figure 4 for a description of the representative voltammetric plots.

**Supplementary Materials and Methods**

Ovariectomy

Female rats (n = 24) were bilaterally ovariectomized (OVX) as previously described (Tanner et al., 2023). Briefly, under ketamine (75.0 mg/kg i.p.) and medetomidine (0.5 mg/kg i.p.) anesthesia, a dorsal incision was made in the skin followed by bilateral incisions through the muscle wall. Ovaries were located, the uterine horns were ligated with absorbable Vicryl sutures (4-0, FS-2), and the ovaries were removed. The muscle and skin incisions were closed with absorbable sutures. Bupivacaine (2 mg/kg) was applied at the sight of muscle incision prior to skin closure. Injections of carprofen (5 mg/kg, s.c.) and penicillin G (22,000 IU/rat s.c.) were administered at induction and every 24 hours for 72 hours after surgery. Rats recovered for 2 weeks prior to experimentation. Vaginal lavage after OVX verified lack of cycling in 100% of OVX.

Conditioned Fear Behavior

Fear conditioning, extinction, and renewal tests were separated by 24 hours, while spontaneous recovery took place 1 week following fear extinction, and occurred in distinct contexts (A, B, C design). Schematics depicting experimental designs are shown in Figures 1A, 2A, 3A and 4A. Freezing was defined as the absence of all movement except that required for respiration and was used as a measure of fear in all behavioral tests. Behavior was recorded with overhead cameras and videos were later scored both with EthoVision XT (Leesburg, VA) and by a human experimenter blind to treatment conditions of the animals. EthoVision was used to quantify locomotor activity prior to the first CS presentation during each behavioral test.

Rats were transported in their home cages to a behavioral testing room to undergo fear conditioning. Rats were placed into custom, rectangular conditioning chambers (context A; 20” W x 10” D x 12” H) with a shock grid floor (Coulbourn Instruments, Allentown, PA). Conditioning chambers were contained within individual sound-attenuating cabinets illuminated by red lights. Rats were allowed 3 minutes to explore the context, after which 4 auditory CS (10 second, 80 dB, 2 KHz), each co-terminating with a 1 second, 0.8-mA foot shock US were delivered on a 1 minute inter-trial interval. Auditory stimuli and foot shocks were delivered through Coulbourn tone generators and shock scramblers controlled via Noldus EthoVision XT software through a custom interface. Rats remained in the conditioning chamber for 1 minute after the last shock before being transported back to the housing room. Conditioning chambers were cleaned with water between rats.

During auditory fear extinction in context B, rats were placed into either a custom Plexiglas chamber that was either rectangular (15” W x 15” D x 20” H) with a textured floor or a triangular (15” sides x 20” H) with a smooth floor. Both chambers were counterbalanced so that half the rats were exposed to fear extinction in the rectangular chambers and the other half in the triangular chambers. Fear extinction took place in the same sound-attenuating cabinets as conditioning. Cabinets contained vanilla scent and were illuminated with bright white lights, while a fan inside the cabinet provided ventilation and background noise. Rats were transported to the sound-attenuating cabinets in their assigned chambers. After a 3 minute exploratory period, the auditory CS was presented 20 times (1 minute ITI) in the absence of the foot shock US. Rats were removed from context B 1 minute after the last auditory CS presentation and returned to their homecages in the housing room. context B chambers were cleaned with 10% ethanol between tests.

During fear renewal testing, half of the rats were re-exposed to the auditory CS in the same context that fear extinction took place (context B; Same) while the other half were re-exposed to the auditory CS in a different context than where fear extinction took place (context C; Different), so that rats that underwent fear extinction in the rectangular Plexiglas chamber were now placed into the triangular chamber, and vice versa. Rats in context B chambers were transported to the sound attenuating cabinets with identical treatment conditions as context B during fear extinction. Rats in context C chambers were transported to the sound attenuating cabinets consisting of a raspberry scent and dimly lit by a lamp located outside of the cabinets. After a 3 minute exploration period, the auditory CS was presented (5-20 CS, depending on experiment; 1 minute ITI) in the absence of the foot shock US. Rats were removed from context B or context C 1 minute after the last auditory CS presentation and returned to their home cages in the housing room. Context C chambers were cleaned with 1% acetic acid between tests. Experimenters transporting and handling rats also differed between contexts, such that unique experimenters were used for each context. In the experiment in which E2 was administered to OVX rats, 5 CS presentations were used during fear renewal testing to avoid floor effects during spontaneous recovery.

During spontaneous recovery, rats were transported in context B chambers to the sound attenuating cabinets with identical treatment conditions as fear extinction. After a 3 minute exploration period, the auditory CS was presented 20 times (1 minute ITI) in the absence of the footshock US. Rats were removed from context B 1 minute after the last auditory CS presentation and returned to their homecages in the housing room. Context B chambers were cleaned with 10% ethanol between tests.

mCherry Densitometry

Immunohistochemistry (IHC) was performed on brain sections from all rats injected with hM4Di and used in behavioral testing. Final group numbers used for densitometry very within and between brain regions due to damage incurred during FSCV, slicing or tissue processing for IHC. These regions contained (from rostral to caudal) the prefrontal cortex (3.7mm to 1.70mm rostral from Bregma), striatum (1.6mm to 0.2mm rostral from Bregma), hippocampus/amygdala (−2.12mm to −4.52mm caudal from Bregma), and substantia nigra (-4.8mm to -6.04mm caudal from Bregma). Sections were rinsed 3 times for 10 minutes using 0.01M phosphate buffed saline (PBS), followed by an overnight incubation at room temperature in 5% blocking solution containing 0.3% Triton X, 0.01M PBS, and 5% normal goat serum (NGS). Sections were then rinsed 3 times for 10 minutes in 0.01M PBS, the sections were placed in 3% blocking solution of 0.01M PBS, 5% NGS, and rabbit anti-mCherry (Abcam, Cambridge, UK, Cat# ab167453) at 1:50,000 overnight at room temperature. Sections were then rinsed 3 times for 10 minutes each in 0.01M PBS, then sections were incubated in 3% blocking solution containing 0.01M PBS, 5% NGS, and Alexa Fluor goat anti-rabbit 594 (Thermo Fisher Scientific, Waltham, MA, Cat# lA-11012) at 1:250 for 4 hours at room temperature. Sections were rinsed 3 times for 10 minutes in 0.01M PBS, and tissue was mounted on slides using deionized water and left to air dry slightly before cover slipping with Prolong Gold with DAPI. Cell counts and densitometry values were captured from at least 3 tissue sections and intensity of mCherry signal above background (densitometry) was calculated as previously described (5).

References

1. Velasco ER, Florido A, Milad MR, Andero R. Sex differences in fear extinction. Neurosci Biobehav Rev. 2019;103:81-108.

2. Velasco ER, Florido A, Perez-Caballero L, Marin I, Andero R. The Impacts of Sex Differences and Sex Hormones on Fear Extinction. In: Milad MR, Norrholm SD, editors. Fear Extinction: From Basic Neuroscience to Clinical Implications. Cham: Springer International Publishing; 2023. p. 105-32.

3. Day HLL, Stevenson CW. The neurobiological basis of sex differences in learned fear and its inhibition. Eur J Neurosci. 2020;52(1):2466-86.

4. Bauer EP. Sex differences in fear responses: Neural circuits. Neuropharmacology. 2023;222:109298.

5. Lloyd BA, Hake HS, Ishiwata T, Farmer CE, Loetz EC, Fleshner M, et al. Exercise increases mTOR signaling in brain regions involved in cognition and emotional behavior. Behav Brain Res. 2017;323:56-67.
